# Spectroscopic and Quantum Chemical Evidence of Amine–CO_2_ and Alcohol–CO_2_ Interactions: Confirming an Intriguing Affinity of CO_2_ to Monoethanolamine (MEA)

**DOI:** 10.3390/molecules29235521

**Published:** 2024-11-22

**Authors:** Sahar Hafizi Yazdabadi, Dmytro Mihrin, Karen Louise Feilberg, René Wugt Larsen

**Affiliations:** 1 Department of Chemistry, Technical University of Denmark, Kemitorvet 206, 2800 Kongens Lyngby, Denmark; 2 DTU Offshore, Technical University of Denmark, Elektrovej 375, 2800 Kongens Lyngby, Denmark

**Keywords:** carbon capture, CO_2_, monoethanolamine (MEA), amines, alcohols, vibrational spectroscopy, neon matrices, molecular association, non-covalent interactions, DLPNO-CCSD(T), local energy decomposition

## Abstract

A recent broadband rotational spectroscopic investigation of the cross-association mechanisms of CO_2_ with monoethanolamine (MEA) in molecular beams [F. Xie et al., *Angew. Chem. Int. Ed.*, **2023**, *62*, e202218539] revealed an intriguing affinity of CO_2_ to the hydroxy group. These findings have triggered the present systematic vibrational spectroscopic exploration of weakly bound amine··CO_2_ and alcohol··CO_2_ van der Waals cluster molecules embedded in inert “quantum” matrices of neon at 4.2 K complemented by high-level quantum chemical conformational analyses. The non-covalent interactions formed between the amino and hydroxy groups and the electron-deficient carbon atom of CO_2_ are demonstrated to lift the degeneracy of the doubly degenerate intramolecular CO_2_-bending fundamental significantly with characteristic observed spectral splittings for the amine··CO_2_ (≈35–45 cm^−1^) and alcohol··CO_2_ (≈20–25 cm^−1^) interactions, respectively, despite the almost identically predicted total association energies (≈12–14 kJ·mol^−1^) for these van der Waals contacts, as revealed by benchmark Domain-based Local Pair Natural Orbital Coupled Cluster DLPNO-CCSD(T) theory. These high-level theoretical predictions reveal significantly higher “geometry preparation energies” for the amine··CO_2_ systems leading to a more severe distortion of the CO_2_ linearity upon complexation in agreement with the infrared spectroscopic findings. The systematic combined spectroscopic and quantum chemical evidences for cross-association between CO_2_ and amines/alcohols in the present work unambiguously confirm an intriguing binding preference of CO_2_ to the hydroxy group of the important carbon capture agent MEA, with an accurate vibrational zero-point energy corrected association energy (*D*_0_) of 13.5 kJ·mol^−1^ at the benchmark DLPNO-CCSD(T)/aug-cc-pV5Z level of theory.

## 1. Introduction

The concentration of CO_2_ in the atmosphere, now exceeding 400 parts per million (ppm), poses a significant challenge as a potent greenhouse gas contributing to global warming and climate change [1]. This rise in CO_2_ levels is primarily driven by human activities such as fossil fuel combustion, deforestation, and industrial processes. Despite global efforts to reduce CO_2_ emissions through renewable energy adoption, energy efficiency improvements, and policy measures, it has become increasingly clear that mitigation strategies alone are insufficient to address this challenge. Consequently, carbon capture and storage technologies have emerged as an important complementary strategy in the efforts against climate change, aiming to directly remove CO_2_ from the atmosphere or prevent its release directly from large-scale industrial sources.

The use of advanced solvents for carbon capture, including biphasic solvents and ionic liquids, has been reported extensively in the literature. Both classes of solvents provide unique advantages in terms of CO_2_ absorption and regeneration efficiency. Biphasic solvents, in particular, are promising due to their phase separation properties at elevated temperatures, potentially lowering the energy demands of CO_2_ regeneration compared to traditional aqueous amines [2,3]. Ionic liquids (ILs) have also emerged as promising candidates for CO_2_ capture due to their high CO_2_ solubility and unique properties, including negligible vapour pressure and recyclability, which qualify them as green solvents. ILs can be customised by modifying their anions or cations, allowing specific tuning of their CO_2_ capture capabilities. Various IL types, such as conventional imidazolium-based ILs, task-specific ILs with amino groups, and amino acid-based ILs, have been explored for enhanced CO_2_ absorption efficiency [4,5,6,7,8,9,10]. For post-combustion CO_2_ capture, several non-amine compounds have so far been studied, such as potassium-based and calcium oxide-based sorbents, biochars, and metal–organic materials [11,12,13,14,15].

Despite these alternatives, CO_2_ capture research predominantly focuses on amine-based technologies due to their ability to react reversibly and their cost-efficiency with CO_2_ [11]. Although some amines such as piperazine have been studied as potential CO_2_ absorbing compounds [16,17,18], the non-volatile alkanolamines containing both a hydroxy and an amino group such as monoethanolamine (MEA) and diethanolamine (DEA), are widely employed as absorbents in CO_2_ capture processes [19,20,21,22]. A blend of different alkanolamines, such as a combination of a primary (MEA) or secondary (DEA) amine with a tertiary amine, e.g., *N*-methyldiethanolamine (MDEA) leverages the strengths of each. Tertiary amines provide relatively higher CO_2_ absorption capacity, while primary and secondary amines offer faster CO_2_ reaction rates. The blend improves both the CO_2_ absorption efficiency and rate, as well as reducing the energy costs for solvent regeneration in the gas treatment [23,24].

Amine gas treating, also known as amine scrubbing, is a technology where the flue gas is passed through an aqueous amine solution, in which the amine absorbs CO_2_ [11,25,26]. It is predicted that by 2030, this method will dominate CO_2_ capture technology in coal-fired power plants [26]. Among the various agents employed for CO_2_ capture in amine scrubbing and other technologies, monoethanolamine (MEA) stands out as the most widely utilised alkanolamine due to its high reactivity and efficiency in capturing CO_2_ from industrial emissions [11,27].

The reaction mechanism of MEA with CO_2_ has been a major topic of many investigations [28]. The formation of zwitterion as an intermediate in the carbamate-producing reaction between MEA and CO_2_ in aqueous solutions has been investigated with quantum chemical and molecular dynamic simulation in several studies [29,30,31]. However, a ^13^C nuclear magnetic resonance study on analyzing the reaction intermediates has shown that the mechanisms of capturing CO_2_ in MEA solutions vary with the CO_2_ loading [32]. Moreover, the conformations of MEA and the zwitterion intermediate appear to play a crucial role in the reaction pathway. For instance, it has been shown that if the acidic hydrogen in the amino group of a zwitterion forms an intramolecular hydrogen bond with the oxygen in the hydroxy group, rather than interacting with nearby water molecules, CO_2_ desorption is more favourable than the deprotonation process [33].

A recent broadband rotational spectroscopic work concerned with the vital cross-association mechanisms of CO_2_ with MEA in molecular beams has investigated the initial stages of the gas-phase nucleation between CO_2_ and MEA with the aid of extensive systematic theoretical conformational sampling [34]. The sub-nanometer-scale aggregation mechanisms involving the complexation of up to four CO_2_ molecules with one MEA molecule under jet-cooled conditions were identified. The subtle competition between the strained directional intramolecular OH⋯N hydrogen bond within MEA and the intermolecular non-directional long-range van der Waals forces associated with the step-wise complexation of CO_2_ was uncovered, revealing an intriguing affinity of the first CO_2_ molecule to the hydroxy group instead of the amino group. These surprising findings are in contrast to the general understanding of the association between MEA and CO_2_ in aqueous solutions [30,31,32,33,35,36] and have triggered the present systematic vibrational spectroscopic exploration of prototypical binary 1:1 weakly bound amine··CO_2_ and alcohol··CO_2_ van der Waals complexes.

Although weak van der Waals forces usually solely give rise to minor complexation spectral shifts of intramolecular vibrational fundamentals, the aim has been to obtain spectroscopic evidence for the vibrational motion of the engaged functional groups aided by high-level quantum chemical modelling. The vibrational spectral signatures of weakly bound van der Waals complexes are normally only within reach experimentally under jet-cooled conditions in very specific and narrow spectral regions covered by sensitive laser spectroscopy approaches [37,38,39,40] but are still achievable with direct absorption in inert doped “quantum matrices” of neon with minor influence of the environment [41] by means of broadband Fourier transform infrared spectroscopy [42,43,44,45] at higher optical densities. The present work demonstrates that among the various potential infrared spectroscopic probes for the association between CO_2_ and alcohols/amines, the lifting of the doubly degenerate bending transition of CO_2_ upon complexation into two components with characteristic observed spectral splittings ≈ 20–45 cm^−1^ (≈3–7%) is a very prominent specific signature for these complexation mechanisms.

## 2. Results and Discussion

### 2.1. Infrared Spectrum for the Weakly Bound Binary 1:1 MEA··CO_2_ van der Waals Complex

**Figure 1:**
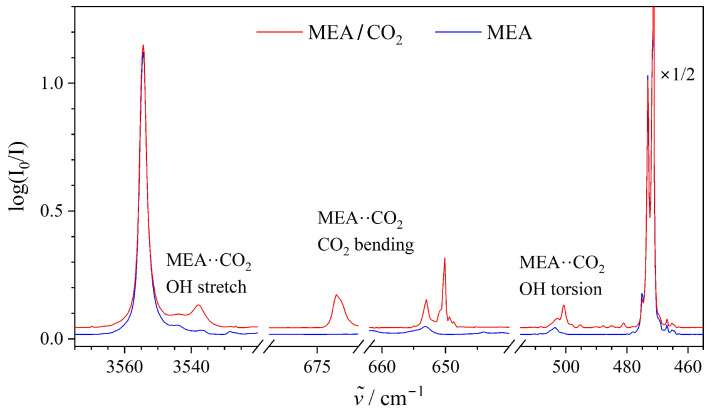
The mid- and far-infrared absorption spectra collected for a MEA/CO_2_ mixture (red trace) and pure MEA (blue trace) embedded in neon at 4.2 K for selected spectral intervals with observed fundamental transitions associated with O-H stretching, CO_2_ bending and large-amplitude OH torsional motion together with proposed vibrational assignments given for the binary 1:1 MEA·· CO_2_ van der Waals complex.

Figure 1 shows the mid-infrared absorption spectra collected for an MEA/CO_2_ mixture embedded in neon at 4.2 K in the relevant spectral regions together with a reference spectrum of pure MEA. The inspection of the complete spectral range has enabled the assignments of new emerging vibrational transitions in the spectral intervals revealing the IR-active fundamental transitions associated with the O-H stretching and large-amplitude OH torsional motion of MEA and the bending motion of CO_2_. The strongly IR-active OH stretching fundamental, assigned to the binary 1:1 MEA··CO_2_ van der Waals complex embedded in neon, is observed at 3538 cm^−1^ and is only slightly red-shifted by 16 cm^−1^ (0.4%) relative to the respective transition for the free MEA monomer reported at 3554 cm^−1^ [46]. A much more significant manifestation of the molecular association is observed for the large-amplitude OH torsional motion of MEA, where this fundamental for the MEA··CO_2_ complex is assigned in the far-infrared part of the spectrum at 500 cm^−1^. This OH torsional fundamental is thereby spectrally blue-shifted by 28 cm^−1^ (6%) relative to the fundamental transition of the free MEA monomer embedded in neon, reported at 472 cm^−1^ previously [46]. The collected spectra do not reveal any new vibrational bands in the vicinity of the previously reported IR-active transitions associated with the asymmetric N-H stretching (3439 cm^−1^), NH_2_ bending (1628 cm^−1^), and NH_2_ wagging (903 cm^−1^) motions of MEA.

These experimental findings provide the first clue that the CO_2_ subunit is not interacting strongly with the amino group, but rather with the hydroxy group of MEA in the cryogenic neon host, as will be supported further when considering the splitting of the doubly degenerate bending fundamental transition of the CO_2_ molecule upon complexation. The double degeneracy of this strongly IR-active CO_2_ bending fundamental is lifted significantly in the binary 1:1 MEA··CO_2_ van der Waals complex resulting in a slightly blue-shifted (by 5 cm^−1^) transition at 673 cm^−1^ associated with the out-of-plane motion and a more significantly red-shifted (by 18 cm^−1^) transition at 650 cm^−1^ associated with in-plane motion with respect to the lone pair-donating atom of MEA. The observed splitting of the CO_2_-bending fundamental in the order of 23 cm^−1^ (3.5%) relative to the fundamental at 668 cm^−1^ reported for free CO_2_ molecules embedded in neon [45] turns out to be a specifically valuable and easily accessible spectroscopic probe for the cross-association mechanisms of CO_2_ with amines and alcohols.

### 2.2. Infrared Spectra for Weakly Bound Binary 1:1 Amine··CO_2_ and Alcohol··CO_2_ van der Waals Complexes

**Figure 2:**
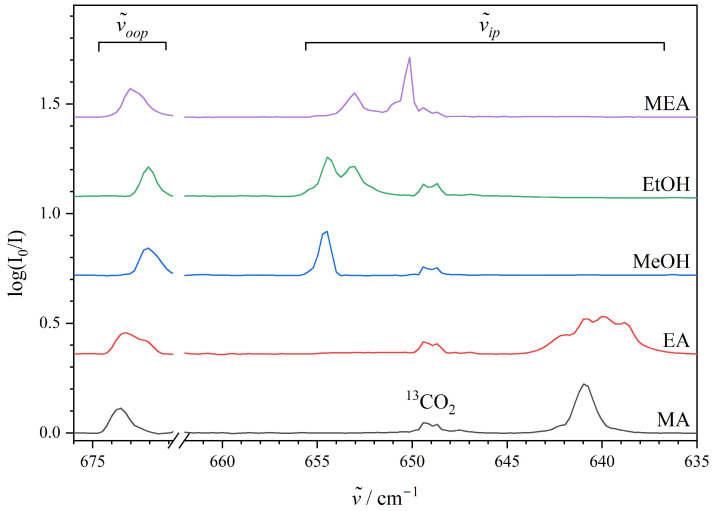
The infrared absorption spectra in the CO_2_-bending region (635–680 cm^−1^) collected for neon “quantum matrices” at 4.2 K doped with mixtures of CO_2_ and methylamine, ethylamine, methanol, and ethanol, and monoethanolamine. The assignments of the red-shifted in-plane (${\tilde{\nu }}_{ip}$) and blue-shifted out-of-plane (${\tilde{\nu }}_{oop}$) components of the CO_2_-bending transition for the binary weakly bound 1:1 van der Waals complexes are indicated together with the absorption from the monomeric bending transition of natural abundant ^13^CO_2_ at 649 cm^−1^ [45]. The narrow region of the strongly saturated absorption from the bending transition of regular monomeric CO_2_ at 668 cm^−1^ [45] has been omitted for clarity.

Figure 2 shows the series of mid-infrared absorption spectra collected for selected amine/CO_2_ and alcohol/CO_2_ mixtures embedded in neon at 4.2 K in the relevant spectral region associated with the CO_2_-bending fundamental transition together with the spectrum collected for the MEA/CO_2_ mixture as a reference. The narrow spectral region of the strongly saturated absorption from the double degenerate bending transition of free monomeric CO_2_ at 668 cm^−1^ and the weak absorptions of (CO_2_)_2_ at 670 and 665 cm^−1^ [45], respectively, have been omitted, whereas the absorption from the monomeric bending transition of natural abundant ^13^CO_2_ at 649 cm^−1^ is observed in all the spectra.

It is evident from inspection of the entire series of spectra that the complexation of CO_2_ with both amine and alcohol compounds systematically is manifested as a lifting of the doubly degenerate bending fundamental of the free monomer into two different bending transitions associated with the out-of-plane motion (the narrow 672–674 cm^−1^ spectral range) and in-plane motion (the more extended 632–651 cm^−1^ spectral range) with respect to the lone pair-donating atom of the complexation partner. The spectral signature observed for the weakly bound 1:1 binary ethylamine (EA)··CO_2_ van der Waals complex is somewhat broad with a distinct sub-band structure assigned to at least three different conformations of the complex with zero-point corrected dissociation energies within 0.7 kJ·mol^−1^ based on the initial CREST conformational analysis, further RI-MP2 optimisations and single-point energies with the benchmark DLPNO-CCSD(T)/aug-cc-pV5Z methodology (Figure S1c–e in Supporting Information). Theoretical predictions employing the same step-wise procedure for the weakly bound 1:1 binary ethanol (EtOH)··CO_2_ van der Waals complex also reveal spectral signatures for two different conformations where the ethanol subunit exists in either the *gauche* or the *trans* conformation (Figure S2b,c) with slightly different band origins for the in-plane CO_2_ bending component (653 cm^−1^ and 654 cm^−1^, respectively, Table 1). The overall trend observed for these spectral signatures of binary 1:1 complexes of CO_2_ with the simplest alcohols methanol (MeOH) and ethanol (EtOH) and the simplest alkylated amines methylamine (MA) and ethylamine (EA) is, however, surprisingly convincing, although the CO_2_-bending fundamentals only provide indirect information about the exact nature of the binding motifs. The out-of-plane CO_2_ bending component turns out to be robustly spectrally blue-shifted by 6–8 cm^−1^ for the entire selection of investigated complexation partners, but interestingly the absolute spectral red-shift of the corresponding in-plane CO_2_ bending component specifically appears to depend more delicately on the chemical nature of the complexation partner. The absolute transition energies for these in-plane CO_2_ bending components assigned for the simplest amine··CO_2_ van der Waals complexes (640–641 cm^−1^) are significantly lower than those observed for the corresponding prototypical alcohol··CO_2_ van der Waals complexes with band origins in the 653–654 cm^−1^ spectral range. These spectroscopic findings provide empirical evidence that the affinity of the CO_2_ molecule to the hydroxy group of MEA is also strongly favoured under inert neon embedding as the observed in-plane CO_2_ bending transition for the MEA··CO_2_ complex is assigned at 650 cm^−1^, close to the findings for the simplest alcohol··CO_2_ complexes.

**Figure 3:**
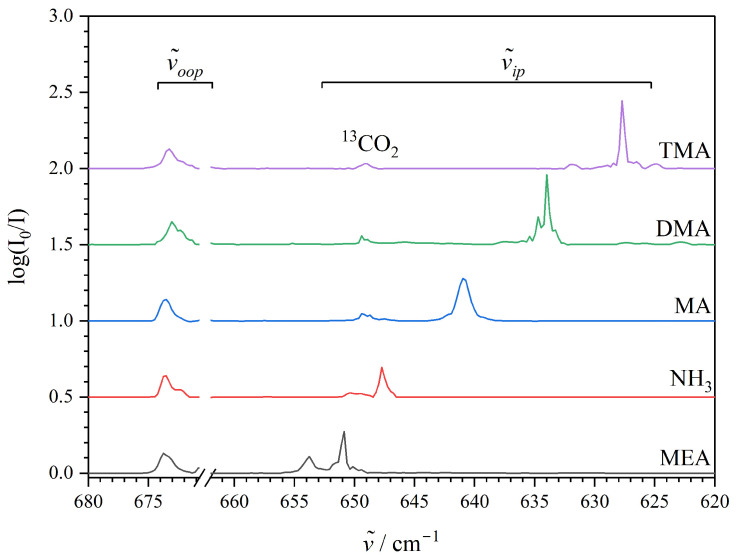
The infrared absorption spectra in the CO_2_-bending region (620–680 cm^−1^) collected for neon “quantum matrices” at 4.2 K doped with mixtures of CO_2_ with monoethanolamine (MEA), ammonia, methylamine (MA), dimethylamine (DMA), and trimethylamine (TMA). The assignments of the red-shifted in-plane (${\tilde{\nu }}_{ip}$) and blue-shifted out-of-plane (${\tilde{\nu }}_{oop}$) components of the CO_2_ bending transition for the binary weakly bound 1:1 van der Waals complexes are indicated together with the absorption from the monomeric bending transition of natural abundant ^13^CO_2_ at 649 cm^−1^ [45]. The narrow region of the strongly saturated absorption from the bending transition of regular monomeric CO_2_ at 668 cm^−1^ [45] has been omitted for clarity.

Before consulting the predictions from theoretical conformational analyses and refined high-level ab initio computations, the pronounced lifting of the degenerate CO_2_-bending fundamental upon the association with amines has been explored further experimentally for an extended set including both ammonia and more alkylated amine compounds. Figure 3 shows the infrared absorption spectra collected for ammonia/CO_2_, methylamine (MA)/CO_2_, dimethylamine (DMA)/CO_2_ and trimethylamine (TMA)/CO_2_ mixtures embedded in neon at 4.2 K in the relevant spectral region associated with the CO_2_-bending fundamental transition. This extended spectral series confirms that the slightly spectrally blue-shifted out-of-plane CO_2_ bending component is basically unaffected by the specific amine complexation partner and is also observed at 673 cm^−1^ for both the binary 1:1 ammonia··CO_2_ and trimethylamine (TMA)··CO_2_ van der Waals complexes. The spectrally red-shifted in-plane CO_2_ bending component of the latter (TMA)··CO_2_ system involving a tertiary amine reveals some sub-band structures indicating multiple conformations of the complex although the dominating transition for the most favoured conformation is easily assigned at 627 cm^−1^. This witnesses the largest splitting of 46 cm^−1^ (6.9%) observed for the degenerate CO_2_ bending fundamental of the entire set of investigated complexation partners (Table 1). The complexation of CO_2_ with the secondary amine, DMA, reveals a less spectrally red-shifted in-plane CO_2_ bending component at 634 cm^−1^ for the most stable conformation, whereas the association with the simplest amine, ammonia, reveals the corresponding transition at 648 cm^−1^ and thereby almost in the vicinity of the observations for the set of alcohol··CO_2_ van der Waals complexes. It is interesting to note that the observed spectral splitting of the degenerate CO_2_-bending fundamental consequently increases almost linearly with the number of methyl groups replacing H atoms on the ammonia molecule in the series of primary, secondary, and tertiary amines as complexation partners (TMA··CO_2_ > DMA··CO_2_ > MA··CO_2_ [47] > ammonia··CO_2_). This observation perfectly correlates with the linear change in the gas-phase basicity of amines with the increase of alkyl substitution, which has been explained by the increase in the polarisability of the molecule [48,49]. These observations will be supported further by the present exploratory quantum chemical predictions.

### 2.3. Quantum Chemical Conformational Analysis of the Weakly Bound Binary 1:1 Amine··CO_2_ and Alcohol··CO_2_ van der Waals Complexes

**Figure 4:**
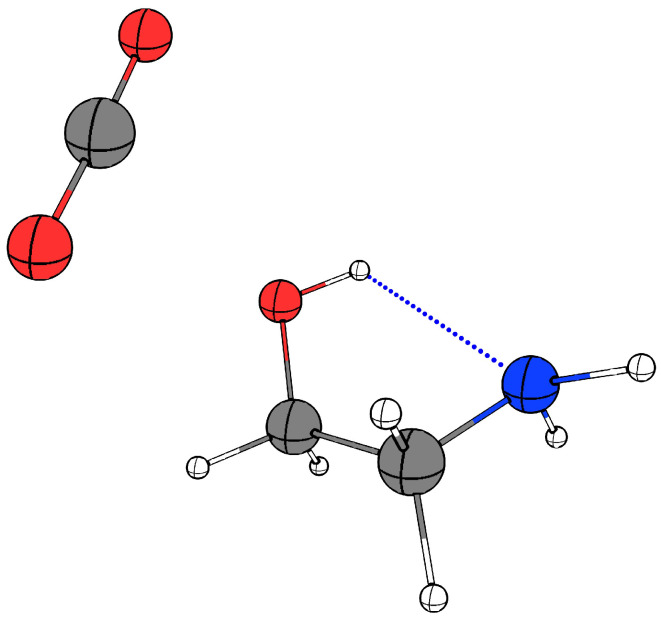
The most stable conformation for the binary MEA··CO_2_ van der Waals complex as reported in Ref. [34] and re-optimised at the RI-MP2/aug-cc-pVQZ level of theory.

The global potential energy minimum geometry of the weakly bound binary 1:1 MEA··CO_2_ van der Waals complex identified via the CREST conformational space exploration tool by Schnell et al. [34], further optimised initially by the B3LYP-D4/aug-cc-pVQZ approach and subsequently employing the RI-MP2/aug-cc-pVQZ methodology, is visualised in Figure 4. In this global minimum structure the intramolecular O–H⋯N hydrogen bond of MEA persist and the C atom of the CO_2_ subunit has established a van der Waals contact with the O atom on the hydrogen bond donating OH group. The refined DLPNO-CCSD(T)/aug-cc-pV5Z electronic energy corrections in combination with the RI-MP2/aug-cc-pVQZ harmonic vibrational zero-point energy corrections provides a dissociation energy *D*_0_ 13.5 kJ·mol^−1^.

**Figure 5:**
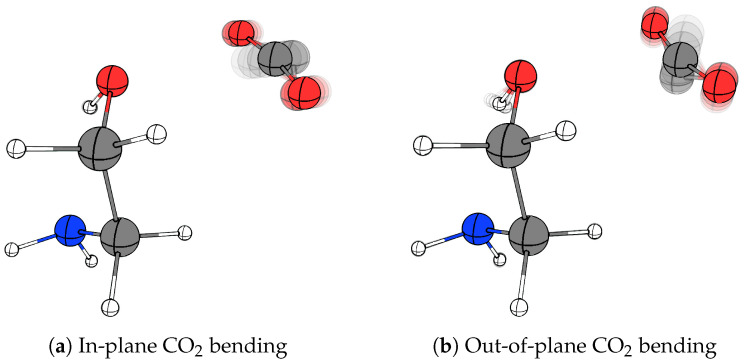
Visualisation of the two components of the CO_2_-bending fundamental for the most stable conformation of the binary 1:1 MEA··CO_2_ van der Waals complex. (**a**) in-line with the lone pair-donating atom (in-plane, red-shifted from CO_2_ monomer) experimentally observed at 650 cm^−1^ (**b**) perpendicular (out-of-plane, blue-shifted from CO_2_ monomer) experimentally observed at 673 cm^−1^.

Table 2 shows the harmonically predicted complexation spectral shifts (Δω = $\omega_{\mathrm{complex}}$−$\omega_{\mathrm{monomer}}$) for the most IR-active O-H stretching, OH torsional, asymmetric NH_2_ stretching, NH_2_ bending and NH_2_ wagging fundamentals together with the predicted splitting of the degenerate CO_2_ bending fundamental for the MEA··CO_2_ van der Waals complex. It is evident that the present observations are strongly supported even by these harmonic vibrational computations at the RI-MP2/aug-cc-pVQZ level of theory. This calculation predicts a harmonic spectral red-shift of 20 cm^−1^ owing to a minor predicted elongation of the O-H bond of 0.001 Å upon complexation (relative to the experimental spectral red-shift of 16 cm^−1^) and accordingly the harmonic calculation predicts a spectral blue-shift of 24 cm^−1^ for the OH torsional fundamental (relative to the experimental spectral blue-shift of 28 cm^−1^) resulting from a slightly stabilisation of the intramolecular O–H⋯N hydrogen bond within the MEA molecule upon complexation with CO_2_ (shortening by 0.02 Å). The agreement between experiment and this harmonic prediction is surprisingly good considering the expected extent of anharmonic character for the latter large-amplitude torsional motion. The fact that the present experimental work does not allow any unambiguous assignments for the perturbed IR-active asymmetric NH_2_ stretching, NH_2_ bending and NH_2_ wagging fundamentals of the binary MEA··CO_2_ complex indirectly supports that the global potential energy minimum predicted and observed by Schnell et al. in molecular beams is also observed in the cryogenic neon “quantum matrix” environment. According to the harmonic vibrational predictions for this most stable conformation, all the spectral shifts of these fundamentals upon complexation should be less than 1 cm^−1^ and thus negligible. Interestingly, the experimentally observed splitting of the CO_2_ bending fundamental of 23 cm^−1^ is also in excellent agreement with the harmonic prediction of 25 cm^−1^ and thereby significant less than observed for the investigated series of amine··CO_2_ van der Waals complexes. The animations of the two predicted (in-plane and out-of-plane) components of the motion associated with the CO_2_ bending fundamental introduced with the complexation are visualised in Figure 5. The unexpected observed dependence of this spectral splitting on the exact intermolecular van der Waals binding motif is explored further by the exploratory theoretical predictions for the entire set of van der Waals complexes.

Table 1 shows the two harmonically predicted components of the CO_2_-bending fundamental for the investigated series of weakly bound van der Waals complexes. In general, the predicted spectral splittings slightly overestimate the experimental values except for the methanol··CO_2_ system, where the smallest observed splitting (18 cm^−1^) is slightly larger than the theoretical (17 cm^−1^) predictions. The theoretical predictions ($\Delta \tilde{\omega }$) strongly support the experimental findings ($\Delta {\tilde{\nu }}_{exp}$) that the splitting of the two different classes of alcohol··CO_2_ and amine··CO_2_ van der Waals complexes falls into two categories; $\Delta {\tilde{\nu }}_{exp}$ ≈ 20 cm^−1^ and $\Delta \tilde{\omega }$ ≈ 20–25 cm^−1^ for the alcohol··CO_2_ systems and $\Delta {\tilde{\nu }}_{exp}$ ≈ 35–45 cm^−1^ and $\Delta \tilde{\omega }$ ≈ 45–55 cm^−1^ for the amine··CO_2_ systems. The splitting of the latter class of systems, however, seems to be more prone to the exact nature of the alkyl substituents. This lifting of the doubly degeneracy of the CO_2_-bending fundamental upon complexation is due to a distortion of the molecule’s linear equilibrium structure as supported by the optimised “distortion angles” and the predicted “geometry preparation energies” for the investigated series of complexes given in Table 1. The distortion angle *∠_distort_*, defined as the difference between the predicted equilibrium O=C=O angle in a specific complex relative to the equilibrium O=C=O angle of 180° for the free non-bonded CO_2_ molecule, is shown to increase significantly when CO_2_ interacts with an amino group relative to the situation when a CO_2_ interacts with a hydroxy group for molecules with the same alkyl substituents although the total predicted interaction energies *D*_0_ are almost identical for the two different classes of 1:1 van der Waals complexes (Table 3). The O=C=O angle is distorted by 1.95–2.16° for the investigated alcohol··CO_2_ systems upon complexation, whereas this angle is distorted by 2.94–3.99° for the amine··CO_2_ systems. The distortion of the O=C=O angle in the most stable conformation of the MEA··CO_2_ van der Waals complex is predicted to be 2.49°, which is only slightly more than for the corresponding alcohol··CO_2_ series (but significantly less than for the amine··CO_2_ series) owing to the additional polarisation of the hydroxy group engaging in the intramolecular O–H⋯N hydrogen bond with the amino group of MEA. The same trend is mirrored in the computed values of the “geometry preparation energy” for the CO_2_ subunit, given for each of the investigated binary van der Waals complexes in Table 1, providing an estimate of the energy “cost” when the CO_2_ subunit is distorted upon complexation. These computed geometry preparation energies show that the the attractive stabilising forces in general are still slightly stronger for the van der Waals contacts between CO_2_ and amino groups than between the CO_2_ and hydroxy groups, although the total theoretical association energies are almost identical at the benchmark DLPNO-CCSD(T)/aug-cc-pV5Z level of theory. The geometry preparation energies are for example computed to 0.31 and 0.67 kJ·mol^−1^ for the methanol··CO_2_ and the methylamine··CO_2_ systems where the total zero-point corrected dissociation energies *D*_0_ are calculated to 12.1 and 11.9 kJ·mol^−1^, respectively. The same trend is observed for the pair of ethanol··CO_2_ and ethylamine··CO_2_ van der Waals complexes of similar size when considering both the computed geometry preparation energies and total zero-point corrected dissociation energies.

## 3. Materials and Methods

### 3.1. Experimental Details

The experimental facility consists of a Bruker Vertex 80V Fourier-transform spectrometer (Ettlingen, Germany) in combination with a customised 4 K closed-cycle cryo-cooler (Model DE-204, Advanced Research Systems, Inc., Macungie, PA, USA). A direct transmission oxygen-free high-conductivity copper window holder attached with a wedged diamond sample window (Diamond Materials, GmbH, Freiburg im Breisgau, Germany) has been mounted onto the cold head of the cryo-cooler [50]. The cryostat is surrounded by a rotatable vacuum shroud equipped with two additional wedged diamond windows together with a gate valve allowing the insertion of the inlet copper tubes into the vacuum space of the cryostat [51]. The wedged diamond windows minimise spectral interference fringes from internal reflections in the windows. The optical arrangement of the spectrometer consists of an air-cooled Globar thermal radiation source, a Ge-coated KBr beam splitter together with a broad-band LN_2_-cooled HgCdTe detector for the complete infrared spectral range (450–4500 cm^−1^). A spectral resolution of 0.6 cm^−1^ has been applied throughout the measurements as the best compromise between the resulting signal-to-noise ratio and sufficient spectral resolution to resolve the observed vibrational sub-band structures.

The doped neon “quantum matrices” are obtained by the simultaneous deposition of neon gas (99.999%, Air Liquide Danmark A/S, Taastrup, Denmark) and co-deposition of specific samples through separate inlet tubes, which are brought to within 5 mm of the cold sample diamond window employing a motorised stage [52]. The neon gas is supplied from a mass flow controller (G-series, MKS Instruments, Inc., Andover, MA, USA) at a flow rate of 6–8 sccm depending on the desired mixing ratio and pre-cooled with a LN_2_-cooled trap (77 K). Methanol (99.9%, Sigma Aldrich, Søborg, Denmark) and ethanol (99.9%, Sigma Aldrich, Søborg, Denmark) vapours were premixed with CO_2_ (99.999%, AGA A/S, Ballerup, Denmark) beforehand, while the samples of the selected amines and CO_2_ were co-deposited separately and flow regulated with fine metering valves. The samples of amines including ammonia (25 wt.%, AnalaR NORMAPUR, VWR Int., Søborg, Denmark), methylamine (40 wt.%, Sigma Aldrich, Søborg, Denmark), ethylamine (66.0–72.0 wt.%, Sigma Aldrich, Søborg, Denmark), dimethylamine (40 wt.%, Sigma Aldrich, Søborg, Denmark) and trimethylamine (25 wt.%, Sigma Aldrich, Søborg, Denmark) were all extracted from the aqueous solutions under vacuum. Pumped and pre-baked molecular sieves (4 Å) have been employed to reduce minor traces of H_2_O in the non-volatile MEA sample (99%, Sigma Aldrich, Søborg, Denmark) while depositing MEA directly from a sublimation vessel thermostated to −7 °C [46]. The combined deposition procedures of neon host and the alcohol/CO_2_ and amine/CO_2_ sample mixtures onto the wedged diamond window were carried out simultaneously for a period of two hours.

Following the acquisition of the initial pre-annealing spectra, the neon “quantum matrices” were gently annealed by raising the temperature to 9.5 K for 60 min. The temperature is regulated by a combination of resistive heaters and silicon diode temperature sensors attached to the window holder and feedback electronics operated by a PID temperature controller (Model 335, LakeShore, Westerville, OH, USA). Subsequently, the heater was deactivated, allowing the sample temperature to relax back to 4–5 K before the collection of post-annealing spectra. The annealing softens the solid neon environment and usually circumvents rare residual matrix site splittings [53,54]. This procedure furthermore triggers the diffusion of molecules within the matrix accelerating the cross-association of CO_2_ and the co-deposited alcohol/amine samples into weakly bound van der Waals cluster molecules. The background spectra were all collected of the fully evacuated cryostat at room temperature at the end of the experiments.

### 3.2. Computational Details

Quantum chemical calculations were performed using the Conformer-Rotamer Ensemble Sampling Tool CREST (version 2.12, Bonn, Germany) [55] and the ORCA quantum chemistry program package (version 5.0.4, Max-Planck-Institut für Kohlenforschung, Mülheim an der Ruhr, Germany) [56,57,58], both installed on the DTU High-Performance Computing (HPC) cluster [59]. The CREST tool with the NCI mode and the GFN2-xTB methodology [60] was employed to generate the most stable conformations of the investigated binary 1:1 alcohol–CO_2_ and amine–CO_2_ van der Waals complexes. For each system, the first 10–15 conformations identified by the CREST utility were selected for further optimisations. These conformations underwent further geometry optimisation and harmonic vibrational frequency calculations using the dispersion-corrected B3LYP-D4 methodology [61,62,63], in combination with the aug-cc-pVQZ basis set and the RIJ-COSX approximation [64,65,66].

Following the B3LYP-D4 optimisation step, further geometry optimisations and harmonic vibrational frequency calculations were repeated using the RI-MP2 methodology [67,68,69], employing the same basis sets and approximations as for the B3LYP-D4 calculations. Finally, the optimised potential energy minima geometries obtained from the RI-MP2 calculations were employed for single-point energy calculations with Domain-based Local Pair Natural Orbital Coupled Cluster with Single, Double, and Perturbative Triple excitations (DLPNO-CCSD(T)) [70,71,72,73,74]. This step involved the aug-cc-pV5Z basis set, together with the RI-JK approximation [75] and tight PNO screening criteria [76] to provide the electronic dissociation energies (*D_e_*) and vibrational zero-point energy corrected dissociation energies (*D*_0_) ensuring highly accurate energy estimations. The geometric distortion of the linear CO_2_ fragment caused by intermolecular interaction has been estimated via the “geometry preparation energy”, which is calculated as the electronic energy difference between the CO_2_ subunit in the distorted geometry as found in the respective van der Waals complex, and the energy of the isolated CO_2_ molecule employing the above-described combination of DLPNO-CCSD(T)/RI-MP2 electronic structure methodologies.

## 4. Conclusions

The present work reports a systematic infrared spectroscopic investigation of a series of weakly bound alcohol–CO_2_ and amine–CO_2_ van der Waals cluster molecules embedded in inert “quantum” matrices of neon at 4.2 K triggered by the recent broadband rotational spectroscopic investigation of the MEA-CO_2_ van der Waals complex in molecular beams [34] revealing (indirectly) an intriguing affinity of CO_2_ to the hydroxy group of MEA. The combined vibrational spectroscopic findings and harmonic RI-MP2/aug-cc-pVQZ quantum chemical predictions help to assign both the strongly IR-active vibrational fundamentals associated with the O-H stretching motion at 3538 cm^−1^ (slightly spectrally red-shifted by 16 cm^−1^ (0.4%) relative to the free MEA monomer) and large-amplitude OH torsional motion at 500 cm^−1^ (spectrally blue-shifted by 28 cm^−1^ (6%) relative to the free MEA monomer) of the MEA··CO_2_ van der Waals complex. The collected spectra did not reveal any bands in the vicinity of the previously reported IR-active vibrational transitions associated with the asymmetric N-H stretching, NH_2_ bending and NH_2_ wagging fundamentals of MEA. The present findings reveal that the non-covalent complexation of CO_2_ with MEA is manifested as a significant lifting of the doubly degenerate CO_2_-bending fundamental of the free molecule into two different components associated with out-of-plane (slightly blue-shifted) and in-plane (significantly red-shifted) bending motion resulting in a spectral splitting of 23 cm^−1^ in accordance with the splitting of 25 cm^−1^ from the harmonic predictions. The out-of-plane CO_2_ bending component turns out to be systematically spectrally blue-shifted by 6–8 cm^−1^ for the entire selection of investigated alcohol··CO_2_ and amine··CO_2_ van der Waals complexes, whereas the absolute spectral red-shift of the corresponding in-plane CO_2_ bending component appears to be specifically dependent on the chemical nature of the complexation partner. Our present findings reveal characteristic spectral splittings for the amine··CO_2_ (≈35–45 cm^−1^) and alcohol··CO_2_ (≈20–25 cm^−1^) interactions, respectively, despite the almost identically predicted total association energies (≈12–14 kJ·mol^−1^) for these van der Waals contacts as revealed by benchmark domain-based local pair natural orbital coupled cluster DLPNO-CCSD(T) theory. The spectroscopic findings provide unambiguous complementary empirical evidence that the affinity of the CO_2_ molecule to the hydroxy group of MEA is also strongly favored in inert neon “quantum matrices”.

## Figures and Tables

**Table 1:** The observed (${\tilde{\nu }}_{exp}$) and predicted harmonic ($\tilde{\omega }$) band origins (cm^−1^) of the in-plane (ip) and out-of-plane (oop) components of the CO_2_ bending transition, the observed ($\Delta {\tilde{\nu }}_{exp}$) and predicted ($\Delta \tilde{\omega }$) spectral splitting (cm^−1^) of the doubly degenerate CO_2_ bending transition, the “geometry preparation energy” $\Delta E_{geo}$(CO_2_) (kJ·mol^−1^) together with the induced distortion angle (*∠_distort_*) away from linearity of the CO_2_ molecule at equilibrium (180°–theoretically predicted O=C=O angle at equilibrium) upon complexation for the present series of binary 1:1 van der Waals complexes. The structural and harmonic vibrational predictions are computed employing the RI-MP2/aug-cc-pVQZ approach and the “geometry preparation energies” are computed employing the DLPNO-CCSD(T)/aug-cc-pV5Z level of theory.

System	${\tilde{\nu }}_{ip}$	${\tilde{\nu }}_{oop}$	$\Delta {\tilde{\nu }}_{exp}$	${\tilde{\omega }}_{ip}$	${\tilde{\omega }}_{oop}$	$\Delta \tilde{\omega }$	$\Delta E_{geo}$(CO_2_)	*∠_distort_*
Methanol··CO_2_	654	672	18	651	668	17	0.31	2.10
Ethanol··CO_2_ (*g*)	653	672	19	643	669	26	0.35	2.16
Ethanol··CO_2_ (*t*)	653	672	19	648	667	19	0.25	1.95
Monoethanolamine··CO_2_	650	673	23	643	668	25	0.47	2.49
Methylamine··CO_2_	641	674	33 [47]	633	677	44	0.67	2.94
Ethylamine··CO_2_	640	673	33	632	675	43	0.71	2.98
Dimethylamine··CO_2_	634	673	39	626	669	43	0.93	3.42
Trimethylamine··CO_2_	627	673	46	612	670	58	1.27	3.99

**Table 2:** Experimental (${\tilde{\nu }}_{exp}$) and RI-MP2/aug-cc-pVQZ predicted harmonic ($\tilde{\omega }$) band origins (intensities given in parentheses, units of km·mol^−1^) for vibrational fundamental transitions associated with the IR-active O-H stretching, OH torsional, asymmetric NH_2_ stretching, NH_2_ bending and NH_2_ wagging fundamentals together with the two components of the CO_2_ bending fundamental of the MEA··CO_2_ van der Waals complex together with the observed complexation shifts $\Delta {\tilde{\nu }}_{exp}$ and the harmonically predicted complexation shifts $\Delta \tilde{\omega }$ (units of cm^−1^).

Mode Description	System	$\tilde{\omega }$	$\Delta \tilde{\omega }$	${\tilde{\nu }}_{\exp}$	$\Delta {\tilde{\nu }}_{\exp}$
OH str	MEA	3748 (76)		3554 [46]	
	MEA··CO_2_	3728 (88)	−20	3538	−16
NH_2_ asym str	MEA	3640 (10)		3439 [46]	
	MEA··CO_2_	3641 (11)	1	-	-
NH_2_ bend	MEA	1652 (30)		1628	
	MEA··CO_2_	1652 (30)	0	-	-
NH_2_ wag	MEA	932 (45)		903	
	MEA··CO_2_	931 (48)	−1	-	-
OH tors	MEA	557 (89)		472 [46]	
	MEA··CO_2_	581 (95)	24	500	28
CO_2_ bend	CO_2_	664 (23)		668 [45]	
	MEA··CO_2_ ip	643 (52)	−21	650	−18
	MEA··CO_2_ oop	668 (26)	4	673	5

**Table 3:** Electronic energies *D_e_*, harmonic vibrational zero-point energy factors ΔZPE and resulting corrected dissociation energies *D*_0_ for the investigated binary alcohol··CO_2_ and amine··CO_2_ van der Waals complexes optimised at the B3LYP-D4/aug-cc-pVQZ and RI-MP2/aug-cc-pVQZ levels together with single-point electronic energies at the DLPNO-CCSD(T)/aug-cc-pV5Z level (units of kJ·mol^−1^).

	B3LYP-D4			RI-MP2			DLPNO-CCSD(T)	
System	*D_e_*	Δ **ZPE**	*D*_0_	*D_e_*	Δ **ZPE**	*D*_0_	*D_e_* ^1^	*D*_0_ ^2^
Methanol··CO_2_	14.3	2.7	11.5	15.1	2.8	12.3	14.9	12.1
Ethanol··CO_2_ (*t*)	14.0	2.2	11.8	15.2	2.2	13.0	14.9	12.7
Ethanol··CO_2_ (*g*)	14.3	2.5	11.8	15.1	2.4	12.7	14.8	12.5
Monoethanolamine··CO_2_	15.5	2.6	12.9	16.8	2.5	14.3	16.0	13.5
Methylamine··CO_2_	15.2	2.9	12.4	15.4	3.0	12.4	14.9	11.9
Ethylamine··CO_2_	16.0	2.8	13.2	16.8	3.0	13.8	15.9	12.9
DMA	16.8	2.7	14.1	18.1	2.7	15.4	16.6	14.0
TMA	18.0	2.6	15.4	20.1	2.2	17.9	17.9	15.7

^1^ Electronic dissociation energies calculated at the DLPNO-CCSD(T)/aug-cc-pV5Z level based on optimised geometries at the RI-MP2/aug-cc-pVQZ level. ^2^ Based on *D_e_*-values at the DLPNO-CCSD(T)/aug-cc-pV5Z level in combination with ΔZPE-values at the RI-MP2/aug-cc-pVQZ level.

## Data Availability

Data are contained within the article and Supplementary Materials.

## Supplementary Materials

The following supporting information can be downloaded at: https://www.mdpi.com/article/10.3390/molecules29235521/s1, The material contains the optimized potential energy minima geometries of the studied alcoholes, amines, and monoethanolamine van der waals complex with CO_2_ in XYZ format, the visualization of the respective structures and the predicted harmonic vibrational mode frequencies for the MEA/CO_2_ complex.
