# Supplementary material for: Spectroscopic and Quantum Chemical Evidence of Amine–CO2 and Alcohol–CO2 Interactions: Confirming an Intriguing Affinity of CO2 to Monoethanolamine (MEA)
Source: Molecules. 2024 Nov 22;29(23):5521. doi: 10.3390/molecules29235521 (PMC11643243; doi:10.3390/molecules29235521)
Supplement: Supplementary file 1 [file molecules-29-05521-s001.zip › molecules-3315699-supplementary.pdf]

SUPPLEMENTARY MATERIAL

Spectroscopic and Quantum Chemical Evidence of Amine-CO_2 and Alcohol-CO_2 Interactions: Confirming an Intriguing Affinity of CO_2 to Monoethanolamine (MEA)

Sahar Hafizi Yazdabadi[1,2], Dmytro Mihrin[1,2], Karen Louise Feilberg[2] and René Wugt Larsen[1]
[1]Department of Chemistry, Technical University of Denmark,
Kemitorvet 206, 2800 Kongens Lyngby, Denmark;
[2]DTU Offshore, Technical University of Denmark,
Elektrovej 375, 2800 Kongens Lyngby, Denmark
Correspondence: rewl@kemi.dtu.dk
Date: 2024

Contents

1 Results from *ab initio* calculations **3**

1.1 Cartesian coordinates of the optimized structures 4

1.1.1 Cartesian coordinates for the Ammonia··CO_2 complex at the RI-MP2 level of theory: 4

1.1.2 Cartesian coordinates for the Methylamine··CO_2 complex at the RI-MP2 level of theory: 4

1.1.3 Cartesian coordinates for the Ethylamine··CO_2 conformations at the RI-MP2 level of theory: 4

1.1.4 Cartesian coordinates for the Dimethylamine··CO_2 conformations at the RI-MP2 level of theory: 5

1.1.5 Cartesian coordinates for the Trimethylamine··CO_2 complex at the RI-MP2 level of theory: 6

1.1.6 Cartesian coordinates for the Methanol··CO_2 complex at the RI-MP2 level of theory: 6

1.1.7 Cartesian coordinates for the Ethanol··CO_2 conformations at the RI-MP2 level of theory: 7

1.1.8 Cartesian coordinates for the Monoethanolamine··CO_2 complex at RI-MP2 level of theory: 7

1.2 The structures of the most stable potential energy minima geometries of CO_2 complexes with alcohols and amines 8

1.2.1 The most stable conformations of amine··CO_2 complexes optimized at the RI-MP2 level of theory 8

1.2.2 The most stable conformations of alcohols··CO_2 complexes optimized at the RI-MP2 level of theory 9

1.3 Vibrational frequencies of the optimized Monoethanolamine··CO_2 complex 10

Chapter 1

Results from ab initio calculations

The Electronic Supplementary Information (ESI) in three main sections provides additional details related to the computational and experimental data presented in the main manuscript.

The first section lists the Cartesian coordinates of the possible conformations of the 1:1 weakly bound van der Waals complexes of the amines, alcohols, and monoethanolamine (MEA) with CO_2. These conformations were identified using the CREST tool, and the geometries were optimized through a series of calculations at the RI-MP2/aug-cc-pVQZ, Def2/J, aug-cc-pVQZ/C, SOSCF, RIJ-COSX, and DefGrid3 levels of theory.

The second section of the following ESI presents the structures of the most stable conformations of the investigated complexes and the structures of more stable conformations for those systems where the experimental data sets revealed several abundant conformations.

The third section contains the calculated harmonic vibrational frequencies, predicted at the same MP2 level for the most stable Monoethanolamine··CO_2 complex.

1.1 Cartesian coordinates of the optimized structures

1.1.1 Cartesian coordinates for the Ammonia··CO_2 complex at the RI-MP2 level of theory:

N	0.585312000	1.926279000	0.081621000
H	1.246664000	1.181047000	-0.086928000
H	-0.072195000	1.908535000	-0.685552000
H	0.070962000	1.678526000	0.915477000
C	1.825239000	4.549656000	0.481136000
O	2.202810000	4.582314000	-0.622144000
O	1.467878000	4.561444000	1.591880000

1.1.2 Cartesian coordinates for the Methylamine··CO_2 complex at the RI-MP2 level of theory:

C	2.075865000	-0.734861000	-0.261469000
N	1.475547000	0.498456000	0.246008000
H	1.798241000	1.291973000	-0.290168000
H	1.766506000	0.666064000	1.199153000
C	-1.365016000	0.063106000	-0.012162000
O	-1.675975000	0.977841000	0.641810000
O	-1.111895000	-0.865377000	-0.673171000
H	1.739235000	-0.897849000	-1.281327000
H	1.719556000	-1.572344000	0.331849000
H	3.167442000	-0.746867000	-0.251635000

1.1.3 Cartesian coordinates for the Ethylamine··CO_2 conformations at the RI-MP2 level of theory:

Conformation 1:

C	1.820534000	0.139629000	-0.624194000
N	0.785813000	1.135272000	-0.338170000
H	0.588724000	1.689690000	-1.161226000
H	1.114854000	1.782174000	0.367541000
C	-1.638968000	-0.333786000	0.094987000
O	-2.191657000	0.516304000	0.671760000
O	-1.135868000	-1.218761000	-0.477524000
H	1.419513000	-0.544894000	-1.369994000
H	2.732805000	0.577582000	-1.040014000
C	2.157236000	-0.625579000	0.639021000
H	1.271566000	-1.119726000	1.030358000
H	2.918244000	-1.378338000	0.446310000
H	2.539944000	0.047799000	1.405431000

Conformation 2:

C	1.556985000	0.440059000	0.241145000
N	0.801908000	-0.591664000	-0.468466000
H	1.075811000	-1.508792000	-0.138958000
H	1.027413000	-0.570706000	-1.455022000
C	-1.979225000	-0.023815000	0.103085000
O	-1.607903000	0.759503000	0.885100000
O	-2.406063000	-0.792072000	-0.664247000
H	1.223112000	1.406824000	-0.133026000
H	1.260963000	0.400962000	1.288306000
C	3.069409000	0.330578000	0.121885000
H	3.379283000	0.394570000	-0.920137000
H	3.566421000	1.128463000	0.671882000
H	3.418643000	-0.621500000	0.518779000

Conformation 3:

C	1.312558000	0.340865000	-0.351153000
N	0.759333000	-0.833309000	0.320917000
H	1.314079000	-1.055987000	1.137687000
H	0.811016000	-1.640443000	-0.287379000
C	-1.827911000	0.202125000	0.980102000
O	-1.293403000	1.058600000	1.566854000
O	-2.412139000	-0.626389000	0.402876000
H	0.684629000	0.549516000	-1.217114000
H	1.199089000	1.187289000	0.324681000
C	2.764256000	0.213146000	-0.786578000
H	2.886073000	-0.615515000	-1.482391000
H	3.109375000	1.121793000	-1.277767000
H	3.406703000	0.028806000	0.073137000

1.1.4 Cartesian coordinates for the Dimethylamine··CO_2 conformations at the RI-MP2 level of theory:

Conformation 1:

H	1.211815000	-1.983841000	-0.734322000
C	1.595476000	-1.188415000	-0.100173000
H	1.248770000	-1.367206000	0.916434000
H	2.690552000	-1.240345000	-0.096252000
N	1.081107000	0.094335000	-0.550570000
C	-1.615417000	-0.053781000	0.168678000
O	-1.265926000	-0.070254000	1.283237000
O	-2.029984000	-0.043855000	-0.921744000
H	1.302376000	0.222678000	-1.528467000
C	1.637932000	1.191893000	0.223067000
H	1.285457000	1.110468000	1.249918000
H	1.291799000	2.140797000	-0.178874000
H	2.734147000	1.197802000	0.241316000

Conformation 2:

H	0.732478000	0.106567000	1.945580000
C	1.224033000	0.713607000	1.189426000
H	0.508290000	1.450561000	0.826405000
H	2.051784000	1.259046000	1.657528000
N	1.639906000	-0.124885000	0.076992000
C	-0.987208000	-0.645917000	-0.777440000
O	-1.184187000	-1.464597000	0.030578000
O	-0.853890000	0.172603000	-1.599475000
H	2.229822000	-0.869610000	0.422010000
C	2.357790000	0.639959000	-0.929359000
H	1.669916000	1.347988000	-1.388078000
H	2.722316000	-0.025072000	-1.707832000
H	3.205522000	1.203908000	-0.522677000

1.1.5 Cartesian coordinates for the Trimethylamine··CO_2 complex at the RI-MP2 level of theory:

N	-0.883257000	-0.000364000	0.055863000
C	-1.430513000	-1.199576000	0.659335000
C	-1.428528000	1.187015000	0.683980000
C	-1.155287000	0.014727000	-1.368526000
H	-1.208764000	-1.207531000	1.724403000
H	-0.973426000	-2.076320000	0.205419000
H	-2.521205000	-1.266456000	0.531066000
H	-0.969708000	2.072182000	0.248531000
H	-1.207247000	1.172348000	1.749082000
H	-2.519056000	1.258523000	0.556784000
H	-0.716346000	-0.865886000	-1.834071000
H	-0.714490000	0.903924000	-1.815609000
H	-2.234366000	0.018038000	-1.583360000
C	1.839317000	-0.000788000	-0.055635000
O	1.879275000	1.166333000	-0.060027000
O	1.879858000	-1.167860000	-0.063038000

1.1.6 Cartesian coordinates for the Methanol··CO_2 complex at the RI-MP2 level of theory:

C	-2.295523000	0.323223000	-0.000247000
O	-1.250246000	-0.641078000	0.000084000
H	-2.920169000	0.240309000	0.888148000
H	-2.920229000	0.239626000	-0.888535000
H	-1.814345000	1.294931000	-0.000636000
H	-1.640885000	-1.516734000	0.000509000
C	1.330081000	0.126015000	-0.000038000
O	1.729740000	-0.969459000	0.000438000
O	0.970427000	1.236457000	-0.000520000

1.1.7 Cartesian coordinates for the Ethanol$\cdot\cdot$$CO_2$ conformations at the RI-MP2 level of theory:

Conformation 1:

H	1.837878000	0.087317000	0.464303000
C	0.902341000	-0.078701000	-0.065178000
H	0.830000000	0.636714000	-0.880033000
H	0.912831000	-1.078843000	-0.489953000
C	-0.264761000	0.080016000	0.875856000
H	-0.195637000	-0.639350000	1.693968000
H	-0.274868000	1.083633000	1.304939000
O	-1.455372000	-0.144637000	0.121396000
H	-2.214092000	-0.043928000	0.700867000
C	-2.159458000	-1.034242000	-2.368417000
O	-3.274615000	-0.967913000	-2.031382000
O	-1.057008000	-1.113556000	-2.740817000

Conformation 2:

C	-2.293230000	0.321750000	-0.872252000
C	-1.783301000	0.091368000	0.533204000
H	-2.716039000	-0.592494000	-1.286561000
H	-1.482430000	0.651602000	-1.516680000
H	-3.071782000	1.082632000	-0.875092000
H	-1.351736000	1.003845000	0.934906000
O	-0.727015000	-0.866025000	0.574335000
H	-2.595797000	-0.214624000	1.194811000
H	-1.078040000	-1.714054000	0.291754000
C	1.694638000	0.173690000	-0.125777000
O	1.203140000	1.232930000	-0.111110000
O	2.222860000	-0.865275000	-0.153910000

1.1.8 Cartesian coordinates for the Monoethanolamine$\cdot\cdot$$CO_2$ complex at RI-MP2 level of theory:

C	1.877319000	0.525891000	-0.439567000
H	1.176593000	0.468222000	-1.270276000
H	2.565527000	1.352960000	-0.632769000
C	1.099413000	0.788515000	0.834061000
H	0.499347000	1.690032000	0.737170000
H	1.793819000	0.925631000	1.669624000
C	-1.954672000	-0.127139000	-0.567370000
O	-2.317518000	-1.202124000	-0.300318000
O	-1.626259000	0.952017000	-0.871530000
O	0.214569000	-0.284540000	1.098919000
H	0.725101000	-1.078530000	0.885654000
N	2.531709000	-0.776919000	-0.307279000
H	3.365041000	-0.704708000	0.260930000
H	2.815049000	-1.141671000	-1.204787000

1.2 The structures of the most stable potential energy minima geometries of CO_2 complexes with alcohols and amines

1.2.1 The most stable conformations of amine··CO_2 complexes optimized at the RI-MP2 level of theory

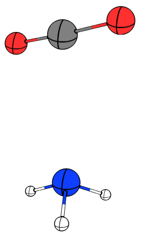

(a) Ammonia··CO_2

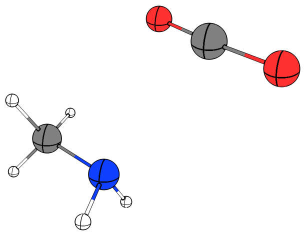

(b) Methylamine··CO_2

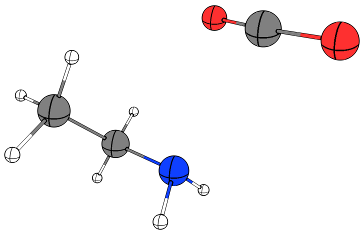

(c) Ethylamine··CO_2
Conformation 1
$\Delta E = 0$

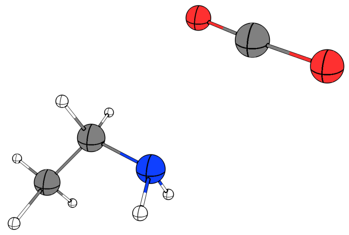

(d) Ethylamine··CO_2
Conformation 2
$\Delta E = 0.3$

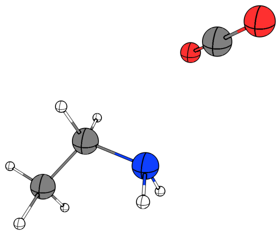

(e) Ethylamine··CO_2
Conformation 3
$\Delta E = 0.7$

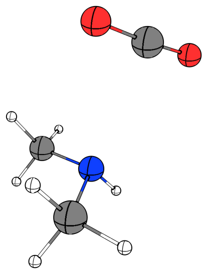

(f) Dimethylamine··CO_2
Conformation 1
$\Delta E = 0$

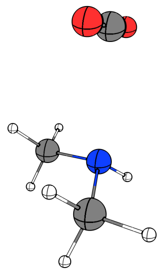

(g) Dimethylamine··CO_2
Conformation 2
$\Delta E = 0.3$

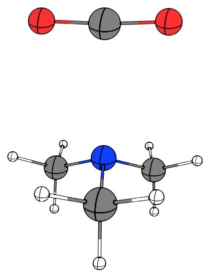

(h) Trimethylamine··CO_2

Figure S1: The most stable conformations of ammonia (a), methylamine (b), ethylamine (c-e), dimethylamine (f and g), and triethylamine (h) van der Waals complex with CO_2 obtained from the CREST conformational search tool and optimized at the RI-MP2/aug-cc-pVQZ level of theory. The ΔE values are calculated based on the zero-point energy corrected dissociation energies D_0 (kJ·mol^{-1}) at DLPNO-CCSD(T)/aug-cc-pV5Z level.

1.2.2 The most stable conformations of alcohols$\cdot\cdot$$CO_2$ complexes optimized at the RI-MP2 level of theory

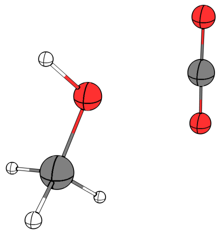

(a) Methanol$\cdot\cdot$$CO_2$

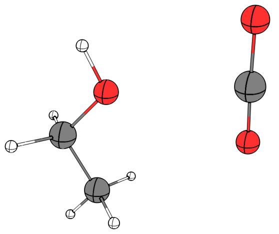

(b) Ethanol$\cdot\cdot$$CO_2$
Conformation 1
$\Delta E = 0$

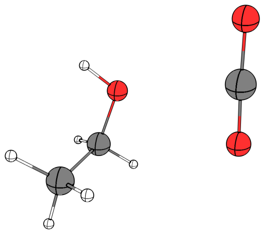

(c) Ethanol$\cdot\cdot$$CO_2$
Conformation 2
$\Delta E = 0.2$

Figure S2: The most stable conformation of methanol$\cdot\cdot$$CO_2$ (a), and the two most stable conformations of ethanol$\cdot\cdot$$CO_2$ van der Waals complex (b) and (c), obtained from the CREST conformational search tool, optimized at RI-MP2/aug-cc-pVQZ level of theory. The ΔE values are calculated based on the zero-point energy corrected dissociation energies D_0 (kJ$\cdot$mol^{-1}) at DLPNO-CCSD(T)/aug-cc-pV5Z level using the prediction for the *trans* conformation of ethanol as the reference.

1.3 Vibrational frequencies of the optimized Monoethanolamine$\cdot\cdot$$CO_2$ complex

Table S1: RI-MP2/aug-cc-pVQZ predicted harmonic vibrational frequencies ($\tilde{\omega}$) and intensities (I) for the most stable conformation of the MEA$\cdots$$CO_2$ complex.

$\tilde{\omega}$	I
33.03	0.82
41.17	0.76
75.45	3.43
103.86	3.94
137.66	1.17
197.27	6.55
262.33	11.73
329.62	1.01
532.85	15.34
581.12	94.72
642.85	52.44
668.27	25.97
821.15	83.65
900.63	10.37
930.53	47.81
1009.46	7.41
1077.42	52.7
1124.34	32.91
1192.19	6.44
1262.63	27.9
1331.66	0.24
1332.11	3.22
1373.26	6.53
1407.33	10.3
1442.57	43.78
1511.84	6.53
1522.2	2.5
1652.47	30.09
2409.84	525.16
3034.02	51.04
3051.64	50.45
3138.44	18.97
3150.43	20.36
3541.67	2.1
3640.7	11.32
3728.39	87.82
